# Prokaryotic viability and active metabolism across a Greenland Sea transect (75°N latitude)

**DOI:** 10.3934/microbiol.2025041

**Published:** 2025-12-11

**Authors:** Alessandro Ciro Rappazzo, Gabriella Caruso, Alessandro Cosenza, Angelina Lo Giudice, Giovanna Maimone, Maria Papale, Manuel Bensi, Vedrana Kovacevic, Maurizio Azzaro

**Affiliations:** 1 Institute of Polar Sciences, National Research Council, Spianata S. Raineri 86, Messina, 98122, Italy; 2 National Institute of Oceanography and Applied Geophysics, OGS, Borgo Grotta Gigante, Sgonico (Trieste), 34010, Italy

**Keywords:** CTC, cell viability, water masses, Greenland Sea, Atlantic, Arctic

## Abstract

In this study, we provided the first comprehensive assessment of prokaryotic viability and respiratory activity across a 75°N transect in the Greenland Sea. Seawater samples collected during the CASSANDRA cruise (early September 2021, Italian Arctic Research Program PRA) were analyzed using LIVE/DEAD BacLight viability staining (L/D) and 5-cyano-2,3-ditolyl tetrazolium chloride (CTC) methods to quantify viable and metabolically active cells, respectively. Total prokaryotic abundance ranged between 0.13 and 8.8 × 10^5^ cells mL^−1^, with metabolically active (CTC+) cells accounting for 0.1–12% of the total. Viable cells accounted for 7–48% of the bacterial community, showing a significant vertical variability that increased with depth (Coefficient of variability 44%), particularly in deeper, nutrient-rich water masses such as the Greenland Sea Deep Water and the Greenland Sea Arctic Intermediate Water, occupying the deep layer (below 2500 m depth) and the intermediate layer (500–2500 m depth), respectively. Significant correlations were found between microbial parameters and environmental variables associated with different water masses, notably nutrients (nitrates and phosphates), whereas temperature showed a more complex, indirect influence. These findings highlight that the prokaryotic community inhabiting the examined transect is well adapted to this extreme marine environment, emphasizing the complex interactions of multiple environmental factors in shaping microbial community structure and activity under low-temperature conditions.

## 1. Introduction

Prokaryotes, including bacteria and archaea, are among the most abundant and metabolically active organisms. In the Arctic Ocean, they are crucial in biogeochemical cycles, such as the degradation of organic matter and the production of greenhouse gases like methane and carbon dioxide [1]. The extreme environmental conditions of the Arctic, such as low temperatures, high salinity, and nutrient scarcity, pose significant challenges to the survival and metabolic activity of these microorganisms [2]. Bacterial metabolic activity plays a critical role within the overall ecosystem functioning, including the remineralization of organic matter *via* the respiration process. Nutrient availability for marine prokaryotes fluctuates spatially and temporally [3],[4], leading bacterial communities to exhibiting a range of physiological states, including active, dormant, and viable but non-culturable (VBNC, [5]). Determining bacterial abundance and distinguishing active and inactive cells can improve the comprehension of the role of microorganisms in dissolved organic matter recycling, as only active cells are the major actors of organic matter turnover [6],[7]. Moreover, understanding the viability and respiratory activity of microbial communities is essential for predicting how their dynamics are modulated in response to environmental changes, such as ice melting, ocean acidification, and increased nutrient inputs from terrestrial sources [8].

In ecological studies, measurements of microbial cell viability and metabolic activity rates are commonly used as proxies to assess microbial metabolism [9]. Bacterial cells can be categorized into three groups: Actively growing cells that contribute to biomass production, living but inactive cells not currently involved in bacterial production but with potential for future activity, and dead or inactive cells that should be considered organic particles [10]. Moreover, microbial metabolism is modulated by several factors, including the availability of organic matter and nutrients, as well as environmental forcings [9],[11]. The commercially available LIVE /DEAD BacLight viability kit (L/D) provides a rapid, simple, and precise tool commonly used to assess the metabolic activity of natural bacterial assemblages; this method quantitatively targets live and dead bacterial cells and yields a measure of total bacterial abundance [12]. It consists of two fluorescent dyes: SYTO 9, which labels all cells with intact membranes, and propidium iodide, which penetrates cells with damaged membranes only. To evaluate the abundance of actively metabolizing bacterial cells, equipped with an active electron transport system (ETS), staining with the redox dye, 5-cyano-2,3-ditolyl tetrazolium chloride (CTC) provides a suitable methodological approach to distinguish active from inactive cells [13]. CTC labeling is used to detect and quantify the metabolic activity of aerobic and anaerobic bacteria; CTC-positive (CTC+) cells are a proportion of live cells showing high metabolic activity and contributing to most bacterial respiration and production, while CTC-negative (CTC−) cells may be viable but exhibit much lower metabolic activity [14]. It is important to note that the LIVE/DEAD and CTC data are not directly comparable, because these methods use different analytical approaches (the first targeting membrane integrity, the second relying on the ability to reduce tetrazolium salts), and, consequently, they provide different information.

Researchers have pointed out that CTC staining protocol is not exempt from potential limitations; criticism to the reliability of this method has been moved in relation to the occurrence of potential biases due to a possible underestimation of the fraction of active cells in natural microbial communities, with consequent inability to detect many functional groups (e.g., fermenting cells, cells with low metabolic rates, or those relying on alternative energy-conserving pathways). Indeed, some metabolically active bacteria may be unstained by CTC due to its toxicity or an inability to reduce CTC under natural aquatic conditions [15]. Additionally, bacterial species may differ significantly in their capacity to minimize tetrazolium salts, and the CTC staining protocol might suppress anaerobic bacterial metabolism, leading to underestimation of active cells [16]. Formazan dye reduction, which may toxic or inhibitory to some microbial cells, could therefore introduce further potential biases, stressing the need to combine different methods/stains to detect metabolically active cells [9]. CTC staining primarily detects cells with an active electron transport system; this means that prokaryotic cells relying on alternative metabolic pathways, such as fermentative or syntrophic taxa, may not be labeled as active. Similarly, slow-growing oligotrophs or cells in dormant states often exhibit low respiratory activity and can be overlooked by this method. This selective detection introduces a bias toward fast-growing, aerobic bacteria, while underrepresenting ecologically important groups adapted to low-energy environments or anaerobic conditions [14],[15]. Indeed, CTC labels primarily cells exhibiting only a strong respiratory activity; this means that functional groups with low or alternative metabolic rates, such as slow-growing or fermentative organisms, anaerobes, and cells using alternative electron acceptors (e.g., sulfate reducers and methanogens) may be underestimated or missed being misclassified as inactive, with consequent biases in interpreting the role of “metabolically active” cells [14],[15]. These methodological biases can distort ecological interpretations by inflating the apparent dominance of aerobic, fast-growing organisms; conversely, taxa involved in fermentation or alternative electron acceptor pathways can remain undetected, leading to an overestimation of aerobic activity and a skewed perception of community vitality. Consequently, models based on CTC data may overpredict carbon turnover rates and misrepresent microbial contributions to nutrient cycling and ecosystem stability. Such inaccuracies can affect assessments of microbial resilience and functional diversity in natural environments, particularly in systems where non-respiratory metabolisms are prevalent [14],[15]. This selective detection can bias interpretations of “metabolically active” cells toward fast-growing, aerobic taxa, while overlooking organisms adapted to low-energy environments. Consequently, ecological conclusions based on CTC data may overemphasize rapid carbon turnover and underestimate the contribution of slow-growing specialists to ecosystem stability and nutrient cycling. Recognizing these limitations is essential for accurate interpretation of microbial community function.

Especially for Arctic samples, controversial results can be obtained using a single method (Live/Dead or CTC); indeed, many cells within the microbial assemblage may be scored as “alive” according to the Live/Dead method; conversely, only a small percentage may be classified as “actively metabolizing” using the CTC staining method. This quantitative discrepancy suggests that most cells may persist in a “survival mode”, becoming ready to recover their metabolic activity as soon as environmental conditions become favorable [1]. Therefore, in the Arctic environment, where many prokaryotes may enter a dormant state like a VBNC state to overcome adverse conditions [2], the combined use of Live/Dead viability staining and CTC labeling is strongly recommended to obtain a more comprehensive picture of the microbial community metabolism [17].

The Greenland Sea, which belongs to the subarctic region, is an important site in the North Atlantic for deep ocean convection, which plays a role in the Atlantic Meridional Overturning Circulation (AMOC) and facilitates the exchange of water masses between the Atlantic and Arctic Oceans, also through the variability in the strength of the Greenland Sea Gyre. The gyre is a large-scale hydrological system, that can modulate large-scale physical and biogeochemical changes in the subarctic marine environment (see [18],[19]). Knowledge on prokaryotic viability and metabolism is fragmentary, remaining limited to some marine and freshwater ecosystems of the Arctic excluding the Greenland Sea [20]–[24]. Based on this knowledge, we aim to provide the first comprehensive assessment of live cells and metabolically active bacteria along a transect crossing the Greenland Sea at 75°N, examining their spatial and vertical distribution, and exploring their association with the water masses identified in the study area. The final objective of this investigation is to get new insights into the metabolic activity of prokaryotic communities in the Greenland Sea.

## 2. Materials and methods

### 2.1. Sampling area and activities

**Figure 1.**
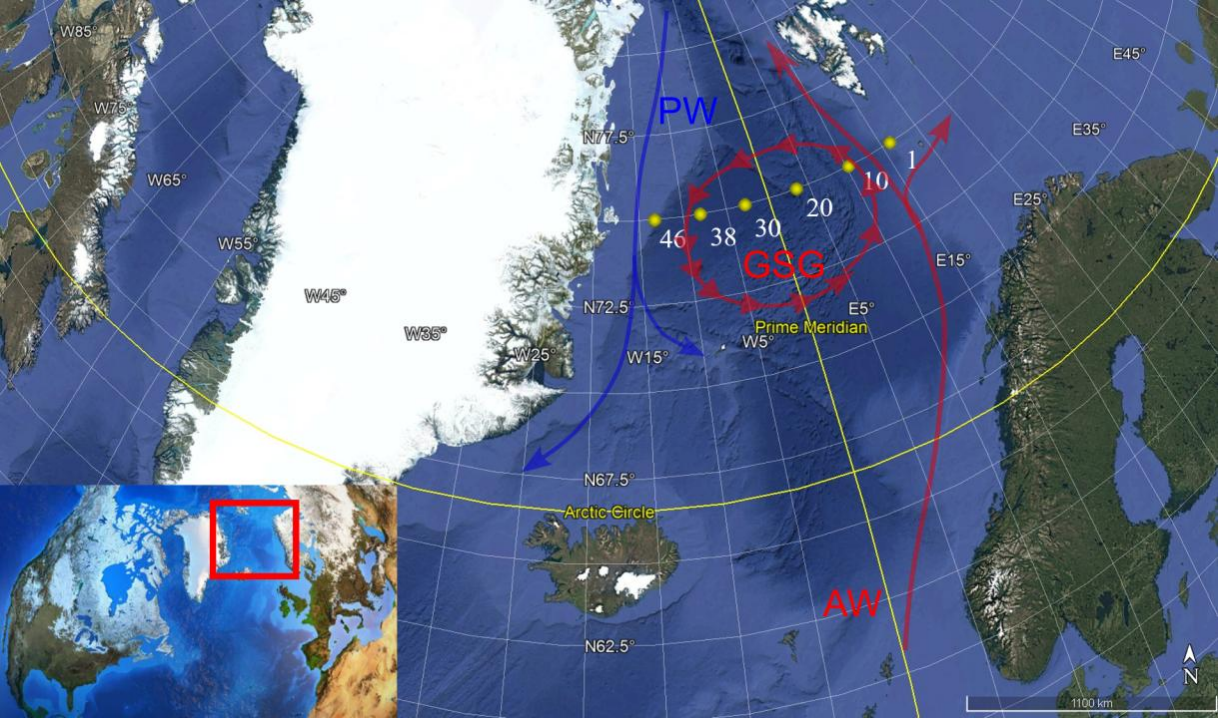
Map of the study area and location of the sampling stations (modified from Google Earth). GSG = Greenland Sea Gyre: PW = Polar Water; and AW = Atlantic Water.

During the CASSANDRA cruise, carried out between 29 August 2021 (Longyearbyen, Svalbard) and 14 September 2021 (Bergen, Norway) (Figure 1), seawater samples were collected on board the icebreaker Laura Bassi (OGS, Trieste), through Niskin bottles (10 L capacity) attached to a conductivity-temperature-depth CTD-Rosette system, used to collect vertical profiles of physical parameters throughout the water column (CTD model Seabird SBE911plus [25]). Here, we do refer to potential temperature (T °C) and practical salinity (S psu) derived from in situ data using the thermodynamic equations for seawater. An additional sensor has been used to collect dissolved oxygen data (SBE43 sensor).

### 2.2. Hydrological characteristics of the sampled stations

The sampled stations encompass a variety of water masses (Figure S1) distinguished by marked gradients in temperature, salinity and dissolved oxygen, particularly in the upper 500 m, due to the presence of Polar (PW) and Atlantic (AW) waters (Figure S2). Nutrient-rich mid-depth and deep layers, with less pronounced gradients, are distinguished by the presence of the Norwegian Sea Deep Water (NSDW), Greenland Sea Arctic Intermediate Water (GSAIW) and Greenland Sea Deep Water (GSDW). Mid-depths across the transect (in the layers 100-500 m on the west side and 500–800 m on the east one) are characterized by waters having transient properties, ascribed to the transition zone (TZ). The hydrological characteristics of the stations in the Greenland Sea are as follows:

Station 1 is at the easternmost end of the transect at the depth of about 250 m. It is largely influenced by the AW, with temperature that ranges between 3.5 and 8.5 °C, and salinity 36.05 and 36.9. The salinity is lower, and temperature is higher in the upper 50 m thick layer, attributed to the surface AW (S-AW), than below.

Station 10 is 185 km west of Station 1, and reaches a depth of about 2500 m. It is influenced by the AW in its upper layer, about 500 m thick, and by the NSDW below 800 m. In between, there is a TZ about 300 m thick.

Station 20 is at the eastern flank of the Greenland Sea cyclonic gyre and has a depth of about 3500 m. It is influenced mainly by the GSAIW, located between roughly 530 and 2130 m, and by the GSDW at larger depths. The S-AW and AW influence its upper 100 m thick layer to a certain extent. In the intermediate layer between 100 and 530 m, there is a TZ.

Station 30 is within the western flank of the gyre and has a depth of about 3600 m. It has characteristics similar to Station 20, but without AW, and with only S-AW influence in its uppermost 50 m.

Station 38 is also within the western flank of the gyre and reaches a depth of about 3300 m. Its hydrographic characteristics are influenced by PW in the surface layer, by the TZ below it, and by GSAIW and GSDW at larger depths.

Station 46, about 220 m deep, is at the westernmost end of the transect, on the continental shelf break. It is influenced by the PW in its uppermost layer. Below it, the TZ extends down to the bottom.

More specifically, the major hydrological characteristics are summarized in Table 1.

**Table 1.** Identification of the major water masses, with indication of the depth range per each sampled station.

	Stations
Water mass	46	38	30	20	10	1
S-AW			surface-50 m	surface-50 m	surface-30 m	surface-50 m
AW				50–100 m	30–480 m	50 m-bottom
PW	surface-50 m	surface-50 m				
TZ	50 m-bottom	50–500 m	50–520 m	100–530 m	480–800 m	
GSAIW		500–2250 m	520–2300 m	530–2130 m		
NSDW					800 m-bottom	
GSDW		2250 m-bottom	2300 m-bottom	2130 m-bottom		

Note: S-AW = Surface Atlantic Water; AW = Atlantic Water; PW = Polar Water; TZ = Transition Zone; GSAIW = Greenland Sea Arctic Intermediate Water; GSDW = Greenland Sea Deep Water; and NSDW = Norwegian Sea Deep Water.

### 2.3. Total prokaryotic abundance

Sample volumes (50 mL) for the determination of total prokaryotic abundance (Bacteria and Archaea) were fixed on board with formaldehyde (Sigma-Aldrich Milano, final concentration 2%) and stored in the dark at 4 °C. Appropriate aliquots (from 2 to 10 mL) were filtered on polycarbonate black membranes (porosity 0.2 µm; GE Water & Process Technologies, Feasterville-Trevose, PA, USA). which were further stained with 4′,6-diamidino-2-phenylindole (DAPI, Sigma, final concentration 10 µg mL^−1^) according to Porter and Feig [26]. Filters were mounted on a glass slide using FA mounting fluid (Zeiss, Milano) and the labeled cells were counted using a Zeiss AXIOPLAN 2 Imaging microscope (magnification: Plan-Neofluar 100× objective and 10× ocular; HBO 100 W lamp; filter sets: Excitation 365 nm, FT395 chromatic beam splitter, emission 445 nm) equipped with an AXIOCAM-HR digital camera. Total abundance was quantified on a minimum of 20 randomly selected microscope fields on two replicate slides to minimize the analytical error related to both the pipetting procedure and the heterogeneous distribution of prokaryotic cells on the filters. When the number of cells detected was very low (generally in 20% of the examined samples), 30–40 random microscope fields were counted. A total of 34 samples were analyzed.

### 2.4. Metabolically active bacteria (CTC+)

The Bac Light Redox Sensor CTC Vitality Kit™ (Molecular Probes) was adopted to quantify actively metabolizing prokaryotic cells. Fresh 50 mM stock solutions of 5-cyano-2,3- ditolyltetrazolium chloride (CTC) were prepared and kept at −20 °C; a volume of 0.1 mL of CTC (final concentration 5 mM) was added to 1 mL of the sample in three replicates, and the samples were incubated at 4 °C in the dark for 5 h; this incubation time was chosen as the optimum after previous trials where incubation for 5, 10, and 18 h was assayed [27]. Incubation was stopped by the addition of filter-sterilised (0.22 µm porosity) formaldehyde (final concentration 2%). The same CTC-labeled slide was used for DAPI counts, with incubation with DAPI (final concentration 2%) after staining with CTC. For each sample, a control was prepared by injecting formaldehyde before CTC addition. Slides were prepared according to the same procedure described for DAPI labeling; cell counts were performed immediately after mounting of the filter using the rhodamine specific filter set (excitation 545 nm; FT580; emission 605 nm). The actively metabolizing cells labeled by CTC (CTC+ cells) could absorb and reduce CTC into a water-insoluble, red-fluorescent formazan product, and were visualized as red-fluorescent cells. At least 100 CTC+ bacteria were counted in >10 microscope fields; the percentage of metabolically active bacteria, as the ratio of CTC-positive (CTC+) to total DAPI-stained cells, was calculated.

### 2.5. Live/Dead (L/D) viability staining method

The viability of prokaryotic cells, in terms of membrane integrity, was assessed through the Live/Dead Bac Light Bacterial Viability Kit™ (Molecular Probes) that uses two solutions of SYTO® 9 (green-fluorescent nucleic acid stain) and propidium iodide (red-fluorescent nucleic acid stain). Stock solutions of each dye were prepared by adding 5 mL of the sample to an appropriate volume of SYTO 9 and propidium iodide, resulting in a final concentration of 6 µM and 30 µM, respectively. The two solutions were mixed thoroughly and incubated at 4 °C in the dark for 1 h. After filtration of 1 mL sample through Nuclepore polycarbonate black membranes (0.22 µm porosity), the same procedure reported above for DAPI staining on sample replicates was followed. Cells were counted under an epifluorescence microscope using the specific sets for SYTO (excitation 475 nm; FT510; emission 530 nm) and rhodamine (excitation 545 nm; FT580; emission 605 nm). Cells labeled by SYTO-9 (“viable”) were visualized as green-fluorescing cells; those labeled by propidium iodide (“dead/non-viable cells”) fluoresced red. To avoid the quick fading of the microscopic field following exposure to the rhodamine filter set, UV- lamp light intensity was lowered below 50%. Cell counts were performed on a minimum of 20 randomly selected microscope fields.

### 2.6. Statistical analysis

Statistical analyses were performed in R (version 4.3.0) and Python (version 3.11) environments using cognate packages and libraries to obtain a robust and complementary data exploration and modelling. In the R software, Spearman correlation (R base package) was applied to assess non-parametric relationships between microbial variables and environmental parameters. Prior to the analysis, physical-chemical datasets including nutrient concentrations were retrieved from Maimone et al. [28]. The nutrient concentrations (NO_3_, NO_2_, NH_4_, and PO_4_) were transformed using the natural logarithm (log(x+1)) to reduce the distortion caused by extreme values and the strong skewness observed in the original distribution. The physical-chemical parameters (temperature, salinity, pH) did not require transformation, as their distributions were symmetrical. The outputs of this analysis were plotted using the corrplot package. Permutational Multivariate Analysis of Variance (PERMANOVA) using the vegan package was conducted to evaluate differences in microbial structure or activity across categories (e.g., water masses). When significant effects were found, Tukey's Honest Significant Difference (HSD) post hoc test (multcomp package) was applied to determine pairwise differences. A Principal Component Analysis (PCA) was also performed with the factoextra package on standardized environmental variables to visualize multivariate structure and identify dominant gradients along the principal axes, potentially representing the main ecological drivers. In parallel, a multiple regression framework was implemented in Python (v3.11), leveraging the statsmodels and scikit-learn libraries. This approach was used to quantify the relationship between the percentage of live cells and key environmental predictors. After testing several model types, Ridge regression was selected for its ability to mitigate overfitting and multicollinearity. Before this statistical analysis, all predictors were standardized. The intercept represents the expected response when predictors are at their mean values Model performance was evaluated using RMSE (Root Mean Square Error) and MAE (Mean Absolute Error), which represent the square root of the mean squared errors and the mean of the absolute errors, respectively, between observed and predicted values.

A non-parametric regression method, the Locally Weighted Scatterplot Smoothing (LOWESS) curve, was applied to the obtained dataset. This method is used to fit a smooth curve to data; it fits simple models (like linear or quadratic polynomials) to localized subsets of the data, using weighted least squares where the nearby points have more influence on the fit than the distant ones. This makes it an effective method for capturing complex trends without assuming a fixed global form.

## 3. Results

### 3.1. Hydrological characterization of the study area

The hydrological structure of the study area was mainly characterized by the identification of five water masses, mentioned in Bensi et al. [19]. The Atlantic Water (AW) occupied the upper layer (0–500 m depth) with temperatures between 3.0 (4.5 °C, at the surface) and 9.0 °C; the Polar Water (PW) occupied the uppermost layer at the westernmost stations with temperature and salinity values typical of melting sea ice (θ < 0 °C, S ≦ 33). The intermediate layer between 100 m and 500 m depth was occupied mainly by a mixed water mass we can define as a transition zone (TZ); conversely, the data in the deep layer below 500 m depth showed the presence of the Greenland Sea Arctic Intermediate Water (GSAIW, −0.9 < θ < 0 °C, S ~ 34.9), the Greenland Sea Deep Water (GSDW, θ < −1 °C, S < 34.9) in the central and westernmost part of the transect and the Norwegian Sea Deep Water (NSDW, θ ~ −0.9 °C, S ~ 34.9) in the easternmost deep part (below 900 m depth) of the transect (Figure S1). Vertical profiles of temperature, salinity, and dissolved oxygen at the stations showed that T reached 8 °C in the subsurface layer in the easternmost part of the transect and dropped below 0 °C at all stations below 500–800 m depth (Figure S2). St. 46 in the westernmost part of the transect was characterized by a PW signal in the upper layer, while AW influenced the easternmost stations (i.e., 01 and 10). Below 800 m depth, the water column appeared to be well homogenized in terms of salinity, while dissolved oxygen decreased with increasing depth, especially below 1300–1500 m depth, where GSAIW, GSDW, and NSDW predominated.

### 3.2. Total prokaryotic cell abundance

**Figure 2.**
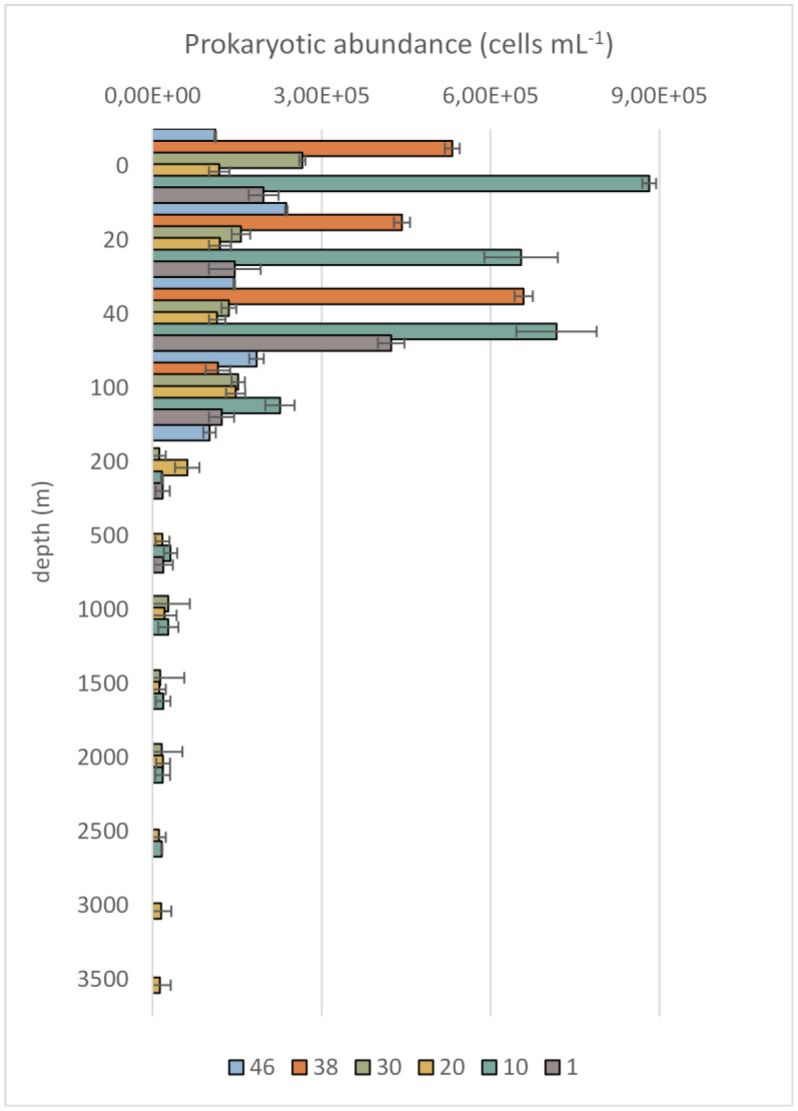
Distribution of the prokaryotic abundance along the water column. The mean value ± standard deviation calculated at each station are reported.

Mean prokaryotic abundance varied between 0.31 and 8.82 × 10^5^ cells mL^−1^, with an average value of 1.67 ± 1.75 × 10^5^ cells mL^−1^. The lowest and highest counts were recorded at St.20 (1500 m depth) and St.10 (5 m depth), respectively. Along the water column, the highest values were recorded in the photic layer, while a progressive decrease was noted with depth (Figure 2). Variability in cell counts among the stations and depths, measured by calculating the coefficient of variability (C.V.), was higher at depths only, where a value of 72% was reached.

### 3.3. Abundance of metabolically active cells

Metabolically active cells (CTC+) from all the collected samples were in the order of 10^3^–10^4^ cells mL^−1^. Among all the examined stations a wide variability was observed (Coefficient of variability = 73%). Overall, CTC+ cells were two orders of magnitude lower than the total prokaryotic ones and accounted for 0.4–12% of the total abundance.

**Figure 3.**
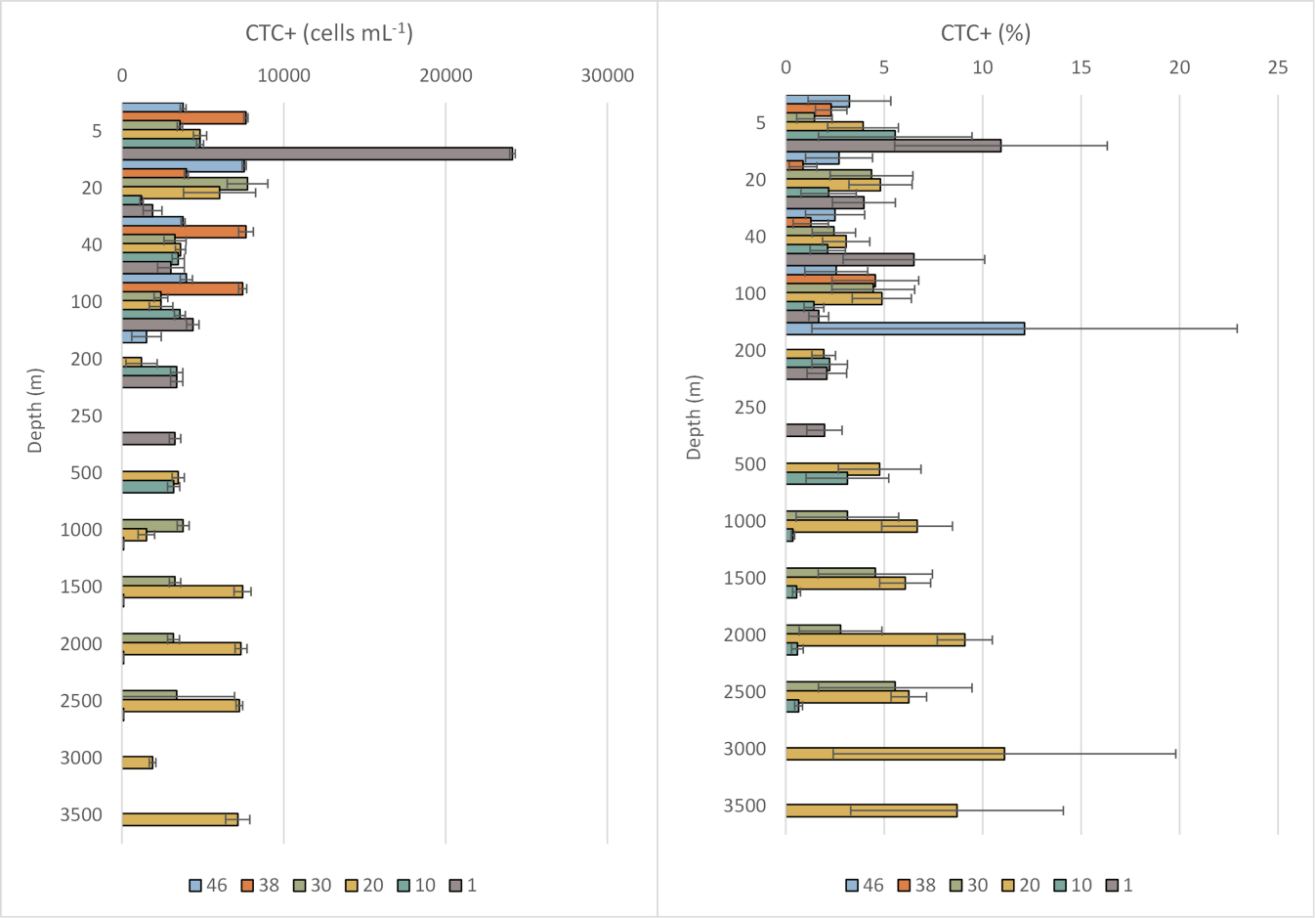
Vertical distribution (mean ± standard deviation) of metabolically active cells (CTC+) as cells mL^−1^ (on the left) and percentage of CTC+ cells to the total prokaryotic abundance at each station (on the right).

In the photic layer (surface to 100 m depth), CTC+ cells were more abundant at St.1 and St.20. In particular, the highest percentage of CTC+ cells was observed at station 1 at 5 m depth (11% of the total prokaryotic counts), followed by St.20, where the percentage of CTC+ cells did not vary significantly along the photic zone. A mean percentage rate of 4.2% of the total was found in AW. In the aphotic layer (200–3500 m depth), CTC+ cells reached their maximum percentage (12%) at St. 46 at a depth of 200 m, corresponding to TZ, and at St. 20, where CTC+ cells showed high percentages in the deeper layers (GSDW; Figure 3). In fact, an increasing trend in CTC+ distribution patterns with increasing depth was reflected by high numbers of CTC+ cells recorded particularly at 2000 and 3000 m depths (9.1 and 11% of the total, respectively).

### 3.4. Abundance of potentially viable cells

The sum of alive and dead cells in all samples fell in the order of 10^4^–10^5^ cells mL^−1^. The number of dead cells was always higher than that of potentially live cells; dead cells (including moribund, dormant, quiescent, not metabolically active cells) contributed on average to 80% of the total L/D counts, while live cells contributed only 20%. Detailed data regarding the quantitative comparison between total DAPI counts and the sum of alive and dead cells are shown in Table 2; this comparison enables checking the accuracy and completeness of the viability assay. Values of the sum Live+dead lower than total DAPI counts suggested that some cells were not classified, and this was presumably due to staining inefficiency, imaging issues, or debris. The highest percentage of live cells (48%) was at St. 46, at 100 m depth, corresponding to PW.

**Table 2.** Comparison between the total prokaryotic (DAPI) *vs* the sum of Live+Dead cell abundance recorded at each sampled station and depth.

STATION	DEPTH (m)	Total DAPI (cells/mL)	Live+Dead (cells/mL)
46	3	1.11E+05	3.23E+04
	20	2.37E+05	3.55E+04
	40	1.45E+05	2.32E+04
	100	1.84E+05	1.29E+04
	200	1.01E+05	2.19E+04
38	2	5.32E+05	1.71E+05
	20	4.43E+05	1.70E+05
	40	6.59E+05	1.68E+05
	100	1.16E+05	1.04E+05
30	4	2.66E+05	1.50E+05
	20	1.57E+05	1.52E+05
	40	1.40E+05	1.40E+05
	1000	1.17E+04	1.17E+04
	1500	1.27E+05	1.27E+05
	2000	1.32E+04	1.32E+04
	2500	1.64E+04	1.64E+04
20	5	1.18E+05	1.18E+05
	20	1.19E+05	1.19E+05
	40	1.15E+05	1.12E+05
	100	1.47E+05	3.99E+04
	200	6.15E+04	3.36E+04
	500	1.76E+04	1.76E+04
	1000	2.12E+04	2.12E+04
	1500	1.17E+04	1.17E+04
	2000	1.87E+04	1.87E+04
	2500	1.13E+04	1.13E+04
	3000	1.51E+04	1.51E+04
	3500	1.29E+04	1.29E+04
10	5	8.82E+05	1.45E+05
	20	6.54E+05	2.01E+05
	40	7.17E+05	1.51E+05
	100	2.26E+05	3.59E+04
	200	1.66E+04	1.66E+04
	500	3.17E+04	3.17E+04
	1000	2.79E+04	2.79E+04
	1500	1.87E+04	1.87E+04
	2000	1.79E+04	1.79E+04
	2520	1.60E+04	1.60E+04
1	5	1.97E+05	1.97E+05
	20	1.46E+05	1.46E+05
	40	4.23E+05	1.33E+05
	100	1.22E+05	8.56E+04
	200	1.78E+04	1.78E+04
	250	1.89E+04	1.89E+04

**Figure 4.**
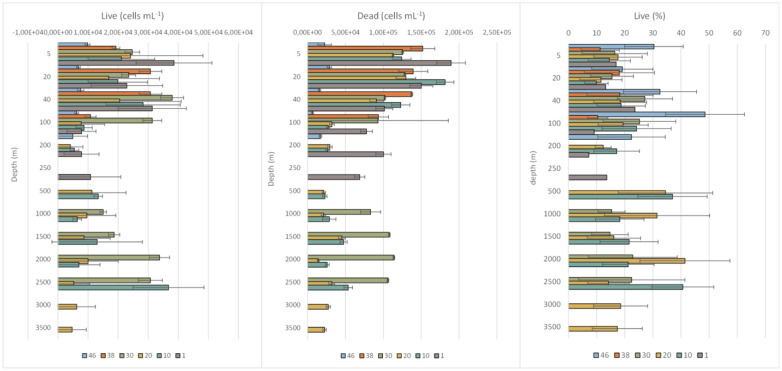
Vertical distribution (mean ± standard deviation) of Live and Dead prokaryotic cells (in cells mL^−1^), and percentage of living cells of the total, calculated at each station.

In the photic layer (surface to 100 m depth), the greatest percentages of live cells (with mean values of 33 and 21%, respectively) were recorded at St.46 and 30, where PW and surface AW were the major water masses. In the aphotic layer (200–3500 m depth), viable cells slightly increased at St.20 and 10 (Figure 4). At St.20, peaks of live cells reaching 34, 31, and 41% of the total were found at 500, 1000, and 2000 m depths, respectively, in correspondence with TZ and GSAIW. Conversely, at St.10, high percentages of viable cells (37 and 41%) were recorded at 500 and 2500 m depths, respectively, in the TZ and NSDW.

### 3.5. Distribution patterns of total prokaryotes and their living and active fractions in relation to water masses

Total prokaryotes reached the highest abundance in PW and AW (St. 38 and St.10, respectively) and thereafter decreased with increasing depth (GSAIW, NSDW, and GSDW). Viable and metabolically active cells were more abundant in colder and deeper waters.

**Figure 5.**
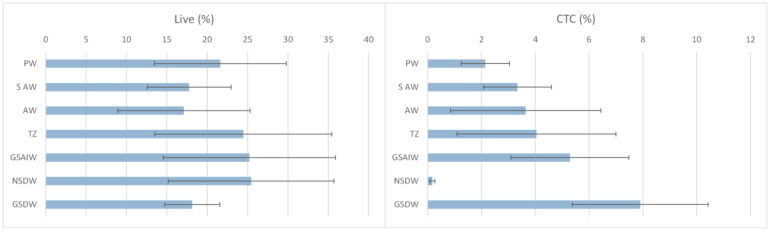
Distribution of Live (left) and actively metabolizing cells (right) in the water masses. PW (Polar Water), S-AW (Surface Atlantic Water), AW (Atlantic Water), TZ (Transition Zone), GSAIW (Greenland Sea Arctic Intermediate Water), NSDW (Norwegian Sea Deep Water), and GSDW (Greenland Sea Deep Water).

The percentage of live cells (Figure 5, left) increased from 17% in AW—occupying the surface water column down to 500 m (St.10 and St.1), to peaks at 25% in the deep layer below 500 m depth, occupied by the Norwegian Sea Deep Waters (NSDW) at the pelagic St.10, and by the intermediate waters of the Arctic Greenland Sea (GSAIW) at St.20 and St.30 located in correspondence of the Greenland Sea Gyre. A further percentage peak of 21% was at St.46 and St.38, where a thin layer of PW was identified.

Metabolically active cells showed an increasing trend from the PW to GSDW, except for the NSDW, where very low percentages were found. CTC+ cells displayed a percentage peak (8%) in the GSDW, followed by a percentage of 5.3% in the GSAIW, at St. 20 and 30 (Figure 5, right).

### 3.6. Statistical analysis

**Figure 6.**
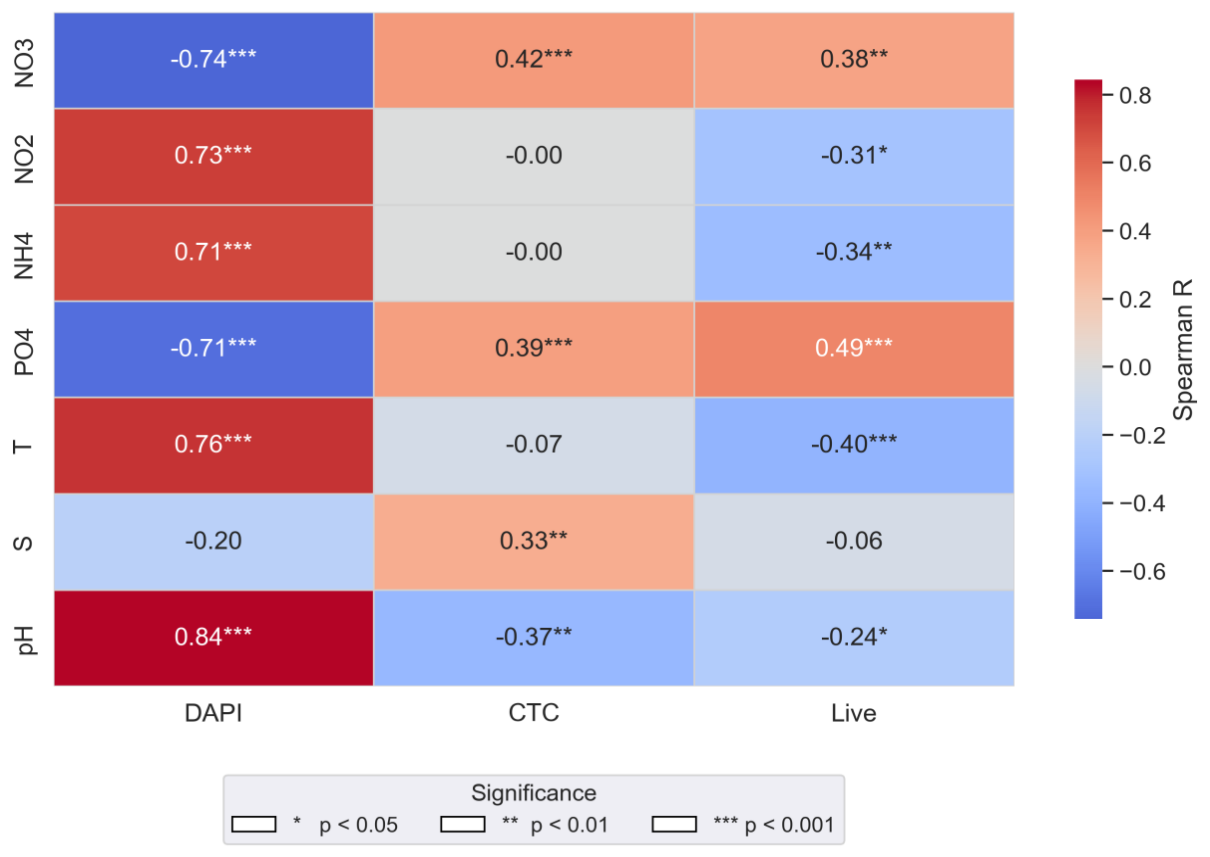
Spearman's correlation matrix between chemical-physical and microbiological parameters (DAPI, CTC, Live).

Spearman's correlation analysis revealed significant relationships between physical-chemical and microbiological parameters along the Arctic water column, indicating that the distribution of different cell fractions is influenced by specific environmental variables (Figure 6). Particularly, the total prokaryotic abundance (DAPI) showed a very strong positive correlation with NO_2_ (r = 0.73, p < 0.001), temperature (r = 0.76, p < 0.001), NH_4_ (r = 0.71, p < 0.001) and pH (r = 0.84, p < 0.001), and significant negative correlations with NO_3_ and PO_4_ (r = −0.74 and −0.71, p < 0.001).

Live cells showed a positive correlation with PO_4_ (r = 0.49, p < 0.001) and pH (r = −0.24 and p < 0.05), while negative correlations were observed with NO_2_, NH_4_, temperature, and a modest but significant correlation with salinity (r = −0.06, non-significant).

Metabolically active cells (CTC+) correlated positively with NO_3_ (r = 0.42 and p < 0.001) and PO_4_ (r = 0.39 and p < 0.001) and showed a weaker but significant correlation with salinity (r = 0.33, p < 0.05), while a significant negative correlation was observed with pH (r = −0.37 and p < 0.01). These results indicated that nutrient concentrations and pH are the major environmental drivers of microbial activity, whereas salinity exerts a minor but detectable effect limited to CTC+ cells (see Figure 6). The different responses observed by total DAPI and live cells with respect to nutrients could be explained by the inclusion of dormant cells, which do not metabolise nutrients, in the total DAPI counts.

The PERMANOVA test was used to analyze the differences related to the variable “water masses”. A pseudo F-value of 18.34 was obtained, indicating that the differences among the groups were highly significant (p < 0.001). This suggested that the variable water masses had a significant effect on the microbial or environmental parameters. The Tukey test indicated that the compared groups were statistically different (Figure S3).

Scatterplots showing the relationships between physico-chemical parameters (NO_3_, NO_2_, NH_4_, PO_4_, temperature, salinity, and pH) and microbial variables (total prokaryotic abundance (DAPI, metabolically active cells), CTC+, and live cells (Live)) are reported in Figure S4. Several associations display non-linear patterns, suggesting threshold effects, saturation, or changing gradients in microbial response to environmental variation. Notably, DAPI-stained cells show strong positive correlations with pH and temperature; a unimodal response is observed with respect to nitrite (NO_2_). Conversely, microbial fractions show opposite trends to phosphate (PO_4_), with a negative association for DAPI cells, a positive relationship for CTC+ ones, and a non-linear, initially increasing, pattern for Live cells, suggesting a weak positive response at low PO_4_ concentrations that tends to level off at higher values. These results indicated that microbial assemblages respond differently to nutrient enrichment, reflecting distinct physiological states within the prokaryotic community.

**Figure 7.**
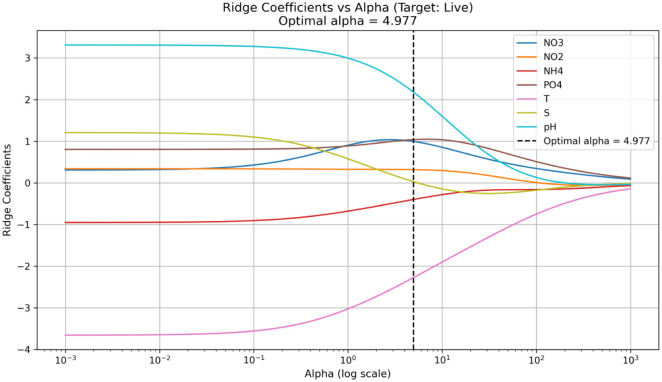
Evaluation of the Ridge regression model built to analyze the relationship between physico-chemical parameters (NO_2_, NO_3_, NH_4_, PO_4_, temperature, salinity, and pH) and the percentage of live prokaryotic cells.

The Ridge regression model (Figure 7) revealed that the abundance of prokaryotic live cells was negatively associated with temperature, positively influenced by pH. Model performance showed that 35% of the variance in cell counts was explained in the training set (R^2^ = 0.35), while the test set retained a moderate predictive ability (R^2^ = 0.16). Although RMSE and MAE increased in the test set, they remained within acceptable bounds, indicating a satisfactory generalization. Overall, the Ridge approach effectively addressed multicollinearity among environmental variables, ensuring more stable and interpretable coefficient estimates (Figure S5).

To better illustrate the impact of regularization on model structure, we plotted the evolution of Ridge coefficients as a function of the regularization parameter α (ranging from 0.001 to 1000 on a logarithmic scale). This graphical representation shows how the weight of each predictor changes as penalization increases. Notably, PO_4_, pH, and NO_2_ retained relatively stable coefficients across the regularization range, suggesting a robust and consistent association with microbial cell viability. In contrast, NO_3_, NH_4_, and salinity coefficients rapidly approached zero, indicating a weaker or less stable influence. It should be noted that this description refers to the asymptotic behavior of the coefficients for very high values of the regularization parameter. For interpretation and selection of the most influential predictors, coefficients obtained only at the optimal value (α = 4.977), highlighted by the vertical line in Figure 7, were considered. In this range, the coefficients represent the balance between penalization and the predictive ability of the model.

**Figure 8.**
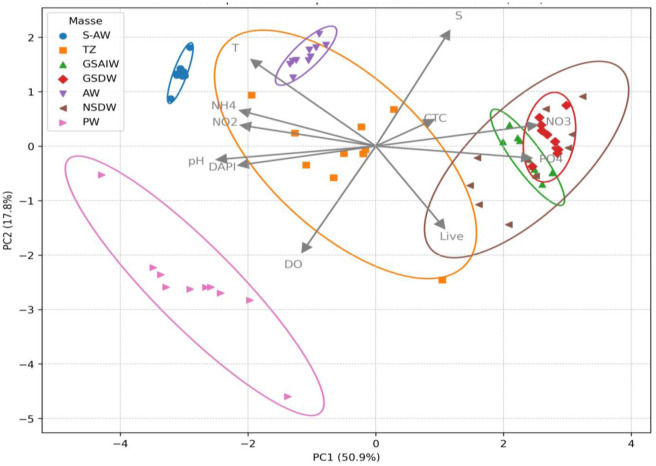
Principal Component Analysis biplot showing the distribution of water mass types (S-AW, TZ, GSAIW, GSDW, AW, NSDW, PW), and environmental variables (T, S, DO, pH, DAPI, Live, CTC, NO_2_, NO_3_, PO_4_, and NH_4_). Ellipse shows the 95% confidence intervals around each group.

PCA outputs showed that the first two principal components (PC1 and PC2) together explained 68.7% of the total variance, with 50.9% and 17.8% explained by PC1 and PC2, respectively (Figure 8). Table 3 reports the contribution (loadings) of each standardized environmental and biological variable to the first two principal components (PC) of the PCA. High absolute loading values indicate variables with the greatest influence on the separation of samples in the PCA biplot.

**Table 3.** Loadings of the first two principal components (PCA biplot with 95% confidence ellipses for water masses).

Variable	PC1 (loading)	PC2 (loading)
T	−0.308	+0.424
S	+0.182	+0.564
NO_3_	+0.400	+0.104
NO_2_	−0.330	+0.102
NH_4_	−0.333	+0.173
PO_4_	+0.384	−0.058
DO	−0.180	−0.516
pH	−0.393	−0.066
DAPI	−0.336	−0.093
CTC	+0.130	+0.118
Live	+0.166	−0.394

The first axis (PC1) represents a deep-surface gradient, distinguishing deep-water samples, rich in nutrients (NO_3_, PO_4_), from surface water samples, characterized by higher temperatures, higher pH values, and total prokaryotic abundance (DAPI). In particular, negative scores on PC1 are associated with cold samples, low salinity, high concentrations of dissolved oxygen (DO), NH_4_, and NO_2_; conditions typical of shallow polar water masses (PW and TZ). Conversely, positive PC1 scores distinguished deep water masses (GSAIW, GSDW, and NSDW), where lower T and S values, greater prokaryotic abundance, and elevated NO_3_ and PO_4_ values were observed, highlighting the key role of hydrographic stratification and nutrient availability in structuring microbial communities.

The PC2 axis, on the other hand, separated samples based on S and DO, with positive loadings for S and negative loadings for DO, and showed a correlation with the presence of metabolically active cells (CTC+). Lower PC2 values were typical of low-salinity, high-oxygenation samples (PW, TZ), which often had a lower percentage of CTC+ cells. In contrast, high PC2 values were associated with more saline and oxygen-poor water masses (NSDW, GSAIW, and GSDW).

Observation of the clusters in the PCA biplot confirmed that the samples were divided into distinct groups, corresponding to the water masses, highlighting the coherence between the environmental parameters (physical-chemical and microbiological) and the hydrographic structures identified in the study area. This result suggested that hydrographic stratification not only drove the distribution of chemical parameters but also played a key role in shaping the composition and functionality of Arctic microbial communities.

Analyzing the arrangement of the microbiological variable vectors revealed that the distribution of metabolically active (CTC+) and viable (Live) cells was influenced by nutrient availability and local chemical-physical conditions. In deep water masses, in particular, a higher frequency of active and viable cells was observed, favored by eutrophic conditions and lower oxygenation compared to surface layers.

In summary, PCA showed that Arctic microbial communities responded mainly to vertical gradients imposed by hydrographic stratification and environmental parameters: Surface waters, more oxygenated and poorer in nutrients, host communities adapted to limiting conditions; on the contrary, deep waters, rich in nutrients and low in oxygen, supported more metabolically active communities.

## 4. Discussion

### 4.1. Prokaryotic viable and metabolically active fractions

Assessing the viability of microorganisms in Arctic and sub-Arctic aquatic environments is crucial for understanding their role in global biogeochemical cycles and ecological interactions, particularly under the low-temperature conditions characteristic of these regions. Microbial communities, in terms of viable or metabolically active cells, have been studied in areas of the Arctic Ocean [23],[29]–[34]. However, to our knowledge, data on total prokaryotes and their viable/active members for several regions of the Atlantic sub-Arctic, such as the Greenland Sea, are rare. In fact, most studies have been conducted in environmental conditions different from those considered in our research. Additionally, we focused on depths significantly greater than those previously examined, making direct comparisons difficult. Our findings showed that prokaryotic abundance is comparable to that in other Arctic ecosystems during summer and spring seasons, respectively [35],[36]. In this study, the viability of the prokaryotic community was assessed using the Live/Dead viability kit, which relies on the ability to distinguish cells with intact membranes from those having damaged membranes. Although DAPI and L/D methods are not directly comparable, quantitative discrepancies among the total microbial community (labeled by DAPI) and the sum of live+dead fractions (by the L/D method) may be explained by the presence of a percentage of cells that possess living attributes other than the intact membrane and, therefore, undetected by the L/D staining method. Indeed, bacterial cells may survive under different physiological states (i.e., quiescent, dormant, and moribund) that may coexist under stressful environmental conditions [37], making the use of a single staining method unsuitable for an accurate estimate of cell viability.

On average, CTC+ cells accounted for approximately 4% of the total prokaryotic abundance, suggesting that the metabolically active population is relatively very low in abundance. Our estimates were lower than the percentage values reported by Howard-Jones et al. [33] and Tammert et al. [29] in the marginal ice zone of the central and northern Barents Sea, respectively, but higher than those observed in the Kara Sea [23], in the Laptev Sea [30], and in marine waters of the Canadian Arctic [22].

Throughout the water column, the total number of prokaryotic cells labeled by DAPI varied significantly only at lower depths, in contrast to the significant variability displayed over the water column by the CTC+ cells (ranging from 0.4 to 12%, with a coefficient of variability of 74%). According to Posch et al. [38], the low percentages of CTC+ cells could reflect cells that are either dormant or have respiration rates below the detection limit. Moreover, Choi et al. [39] and Howard-Jones et al. [33] reported that a significant fraction (25–90%) of prokaryotes in seawater might be dormant or have very low metabolic activity. In cold environments, microorganisms experience metabolic limitations and slowed cellular processes due to low temperatures [40]. Cold-adapted microbes have evolved strategies, such as adaptations in membrane composition, enabling survival in freezing conditions. A common mechanism involves an increased proportion of unsaturated fatty acids and branched-chain fatty acids, accompanied by a decrease in saturated fatty acids, to maintain membrane fluidity while avoiding rigidity [2],[41]. Dormancy helps polar microbes withstand extended periods of nutrient scarcity and low temperatures. In addition, cold-adapted microorganisms remain metabolically active at subzero temperatures thanks to biofilm formation, cryoprotectants production and enzymatic and membrane adaptations [2],[42]. Understanding these survival mechanisms is critical for microbial ecology research in extreme environments and has practical implications in bioremediation and cryopreservation [42]. Variations in bacterial morphology and respiration are influenced by life-cycle stage or physiological responses to environmental stressors such as grazing and starvation [38].

In most marine environments, the abundance of CTC+ cells ranges from less than 5% in oligotrophic waters to more than 30% in productive estuarine areas, but it rarely exceeds 10% of the total microbial community [10]. However, Smith [43] documented a notably higher percentage (47%) of metabolically active cells in Chesapeake Bay. Comparison of quantitative data is frequently challenging due to the lack of standardized methodologies. The literature emphasizes significant variations in CTC staining protocols, which can considerably affect cell counts [10],[15]. Particularly, the major concerns address the CTC concentration, as the highest numbers of CTC+ cells are observed at CTC concentrations of 2.5 to 4 mM, as well as the incubation time (usually 8 hours or longer for oligotrophic samples [10]). Consequently, depending on the environments and cell types, optimization of the staining kinetics and tetrazolium concentrations are suggested [9],[15]. Caution must be taken when interpreting data, because staining approaches could lead to potential biases, resulting in an underestimation of damaged but metabolically active cells, underestimation of functional groups, e.g., bacterial cells fermenting at low metabolic rates or those relying on alternative energy-conserving pathways that could not be detected by CTC assay based on respiratory activity through electron transport chain, or misclassification due to membrane integrity artifacts. These potential variability sources or methodological limitations could result in underestimates of cell metabolic activity in natural samples, affecting the correct ecological interpretation of the obtained data.

Despite ongoing controversies, the CTC method provides valuable insights into bacterial activity and physiological state [23],[29]. A considerable percentage of prokaryotes in marine ecosystems are either dead or inactive [33]. In the Greenland area examined in this study, dead cells remained spatially constant across the transect, representing most (approximately 80%) of the total community. In comparison, viable cells accounted for the remaining fraction (20%), which agrees with Tammert et al.'s [29] findings. This suggested the occurrence of not-well known microbial mechanisms, which affect the physiological state of prokaryotic communities [29]. The relatively low percentage of viable (both intact and metabolically active) cells may result from predation by bacterivorous flagellates, which preferentially consume metabolically active cells, and osmotic shock-induced lysis due to salinity fluctuations [44]. Other factors, including viral lysis, nutrient limitation, or methodological biases in viability staining, might explain this finding. Bacterial mortality could increase, especially during spring and summer, due to high UV radiation and excretion of bacteriostatic compounds by cyanobacteria and chlorophytes during algal blooms, resulting in seasonal fluctuations in live and dead bacterial abundance [44]. Knowledge of the physiological state of prokaryotic cells, whether they are actively growing, dividing, or viable, is critical for determining their ecological roles. This biological community likely includes dividing, virus-infected, or non-growing viable cells [33].

Marine microbial communities typically exhibit vertical stratification, and depth frequently emerges as a significant factor influencing community composition [45]. A clear association was observed between microbial parameters and water masses in our study. Prokaryotic abundance exhibited a linear decreasing trend across water masses, with notably low values below a depth of 200 m. Interestingly, the distribution of CTC+ cells revealed higher abundances below 500 m, specifically in layers occupied by GSAIW, GSDW, and NSDW and in the intermediate TZ. These vertical patterns suggested adaptations within the Arctic prokaryotic community, enabling its survival under decreasing temperatures, increasing salinity, and reduced oxygen levels. Significant correlations were observed between microbiological parameters and environmental variables, although the causal relationships remain unclear, which agrees with other statements [30]. CTC+ cells correlated positively only with NO_3_ and PO_4_. Unlike other studies, which revealed a strong relationship between metabolically active bacteria and temperature [20], our investigation revealed that temperature does not significantly affect the distribution of this cell fraction, confirming an observation by Paoli et al. [10]. In our Arctic transect, this finding was probably due to the narrow variability of thermal values in these cold marine waters. Across the Greenland transect, the abundance of live cells correlated inversely with temperature, contrasting with the findings of Zampino et al. [46], who reported a positive association between live cells and temperature. A positive correlation between maximum growth rates and optimal temperatures has been reported in psychrophilic and mesophilic prokaryotes [47], suggesting a common underlying mechanism linking short-term physiological responses and long-term evolutionary adaptations. Our findings indicated that in sub-Arctic marine environments, low temperatures limit the expression of metabolic activity in prokaryotes. Dissolved organic matter availability may partially mitigate this limitation by enabling the selection of taxa with metabolic capabilities adapted to harsh conditions. Although temperature remains a fundamental factor influencing microbial life, its role in shaping community composition in our study remains unclear, suggesting that additional environmental factors may influence microbial community distribution.

### 4.2. Statistical elaboration of the dataset

LOWESS curves provided a more detailed visualization of the relationships between environmental and biological parameters, offering an essential complement to linear correlation analyses. In particular, the relationship between pH and total prokaryotic abundance (as indicated by DAPI cells) was strongly positive and consistent both statistically (r = 0.84) and in the curve profile, confirming a favorable influence of pH on microbial development. However, in other cases, the LOWESS representation highlighted more complex trends: For example, the relationship between NH_4_ and DAPI showed an initial increase in biomass up to approximately 0.4 µM, followed by a decrease, suggesting a possible threshold effect. This type of information does not emerge clearly from a linear correlation, highlighting the importance of graphical approaches that enable us to capture nonlinear ecological dynamics.

The evidence of nonlinear relationships and the presence of collinearity between nutrient variables (such as NO_3_, NO_2_, and NH_4_) motivated the use of Ridge regression, which is particularly suited to addressing these issues. The model enabled a more stable estimate of the effect of individual predictors on the percentage of viable cells, thereby reducing the risk of overfitting and enhancing the robustness of interpretation. The nonlinear profiles highlighted by the LOWESS curves further justify the adoption of a penalized approach, capable of capturing real trends in the presence of multivariate and potentially noisy data.

Some relationships between environmental and biological variables showed trends consistent with the presence of ecological thresholds or saturation effects. The response of DAPI cells to phosphate (PO_4_), for example, showed an initial increase followed by a decline above 0.8 µM, suggesting that high phosphorus levels may not correspond to a further increase in biomass. Similarly, the response of live cells to temperature showed a decreasing trend, with a clear negative impact above 2–3 °C. These dynamics may reflect the physiological limits of microbial communities or less favorable environmental conditions, reinforcing the idea that environment-biota relationships should be interpreted ecologically rather than exclusively statistically.

The integration of statistical analyses, penalized regression models, and nonlinear visual representations enable a more complete understanding of the interactions between environmental variables and microbial communities. The LOWESS curves highlight trends that would have been missed by a purely linear interpretation, revealing biologically relevant thresholds, plateaus, and trend reversals. Furthermore, Ridge regression offers an effective solution to multicollinearity management, producing more reliable and interpretable estimates. Together, these tools provide a more realistic and detailed representation of microbial dynamics in the different hydrological contexts observed.

PCA points out that Arctic water masses host distinct microbial communities in this context, highlighting critical environmental drivers that influence microbial distribution and physiological states [48],[49]. A particularly interesting finding is the detection of high percentages of Live and CTC+ cells in association with deeper water masses, such as GSDW, GSAIW, and NSDW, and nutrient-rich conditions (especially nitrates, NO_3_, and phosphates, PO_4_). This suggests that microbial adaptations to deeper, colder environments characterized by increased nutrient availability and decreased DO concentrations take place in the examined transect. Such adaptations potentially involve metabolic strategies optimized for nutrient utilization under low T and DO availability [50],[51]. In contrast with the other water masses, PW, characterized by high DO levels and relatively low nutrient concentrations, cluster separately and host microbial communities adapted to oligotrophic conditions, possibly utilizing strategies such as dormancy or slower metabolism to cope with limited nutrient availability [52].

Within PCA, ammonium (NH_4_), nitrite (NO_2_), pH, and total prokaryotic abundance (DAPI) identify separated clusters mainly due to different water masses, suggesting that these parameters do not exclusively drive community differentiation but rather are shared among water masses. These associations provide valuable insights into the complex interactions between water masses, environmental variables, and microbial assemblages. Our observations can therefore help us understand the Arctic Ocean functioning, especially during the summer sea-ice melting period, which agrees with Vipindas et al. [49].

Globally, the results of statistical analyses outline a picture of differentiated adaptation within the prokaryotic community. Total cells respond to surface parameters related to primary production, and viable cells are preferentially distributed in deep, stable layers. Furthermore, the metabolically active fraction is concentrated in areas rich in oxidized nutrients, typical of deep water masses. The set of correlations, integrated with the stratification visible in the PCA biplot, indicates that Arctic microbial functionality is the result of adaptive strategies involving cellular physiological states, in response to changing chemical and physical conditions and nutrient availability throughout the water column.

## 5. Conclusions

This study provides novel data on the abundance and distribution of total prokaryotes and their living, dead, and metabolically active fractions across an Atlantic sub-Arctic marine transect at 75°N, crossing the Greenland Sea Gyre. Significant microbial adaptations to low temperatures and nutrient limitations were observed in this region. The CTC staining method proved to be a valuable tool for assessing the level of metabolic activity of the microbial assemblage. The low percentage of CTC+ cells in the examined samples suggested that highly metabolically active cells are present in small numbers, indicating a predominance of inactive or dormant cells. Although caution should be taken to interpret these data, due to the potential biases of the CTC method especially in environments where alternative energy metabolisms (such as fermentation) predominate, this result provides novel information for the examined area. Microbial communities exhibited clear relationships with water masses: Nutrient-rich deeper layers host higher percentages of metabolically active cells. Furthermore, oligotrophic PW exhibited microbial communities that are cold-adapted (dormant and with reduced metabolic rates). Environmental variables, particularly nutrients, such as nitrates and phosphates, significantly influence microbial distribution and activity, whereas temperature has a more complex and less evident effect. These findings underscore the importance of multiple environmental factors that interact reciprocally in shaping microbial ecosystems in the Arctic and the complexity of understanding how microbial assemblages respond to ongoing climatic changes. In the future, researchers will need to focus on improving the methods of microbial quantification to better adapt them to the unique conditions of the Arctic regions and on the use of advanced technologies, such as metagenomics or complementary approaches, including BONCAT, which tracks protein synthesis [9], to detect a broader spectrum of cell activity, combined with flow cytometry or Fluorescent-in situ Hybridation, and to correlate cell viability with the genetic and functional diversity of microbial communities. Studies on the prokaryotic community viability and metabolism could also benefit from distinguishing free-living and particle-attached microbial communities within water masses. Such a discrimination would enhance our understanding of the microbial carbon pump, enabling us to evaluate the distinct roles that these communities play in carbon export and sequestration in Arctic ecosystems.

## Use of AI tools declaration

The authors declare they have not used Artificial Intelligence (AI) tools in the creation of this article.


