# Supplementary material for: Prokaryotic viability and active metabolism across a Greenland Sea transect (75°N latitude)
Source: AIMS Microbiol. 2025 Dec 11;11(4):921–45. doi: 10.3934/microbiol.2025041 (PMC12782940; doi:10.3934/microbiol.2025041)
Supplement: Supplementary file 1 [file microbiol-11-04-041-s001.pdf]

Microbiology

AIMS Microbiology, 11(4): 921–945.
DOI: 10.3934/microbiol.2025041
Received: 05 September 2025
Revised: 21 November 2025
Accepted: 01 December 2025
Published: 11 December 2025

https://www.aimspress.com/journal/microbiology

Research article

Prokaryotic viability and active metabolism across a Greenland Sea transect (75°N latitude)

Alessandro Ciro Rappazzo[1], Gabriella Caruso[1,*], Alessandro Cosenza[1], Angelina Lo Giudice[1], Giovanna Maimone[1], Maria Papale[1], Manuel Bensi[2], Vedrana Kovacevic[2] and Maurizio Azzaro[1]

[1] Institute of Polar Sciences, National Research Council, Spianata S. Raineri 86, Messina, 98122, Italy
[2] National Institute of Oceanography and Applied Geophysics, OGS, Borgo Grotta Gigante, Sgonico (Trieste), 34010, Italy

* **Correspondence:** Email: gabriella.caruso@cnr.it; Tel: +390906015423; Fax: +39090669007.

Supplementary

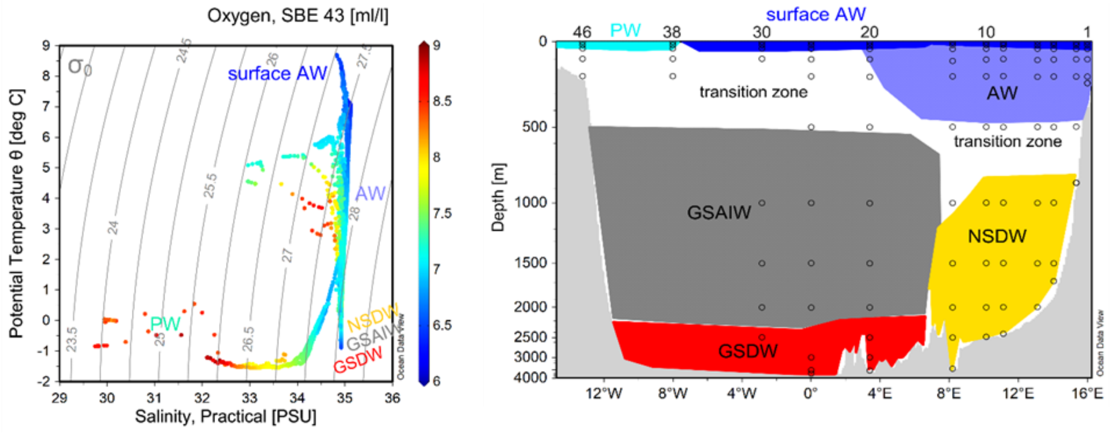

Figure S1. Left panel: Potential temperature-salinity scatter diagram from all the CTD casts carried out along the 75 °N section in 2021 (see black dots on the map in S1b); right panel: Schematic of the vertical distribution of the water masses along the 75°N transect. Major water masses are indicated according to the definitions found in literature [AW: Atlantic Water; GSAIW: Greenland Sea Arctic Intermediate water; NSDW: Norwegian Sea Deep Water; GSDW: Greenland Sea Deep Water; PW: Polar Water or Melting Water]. Figures created with Ocean Data View [1].

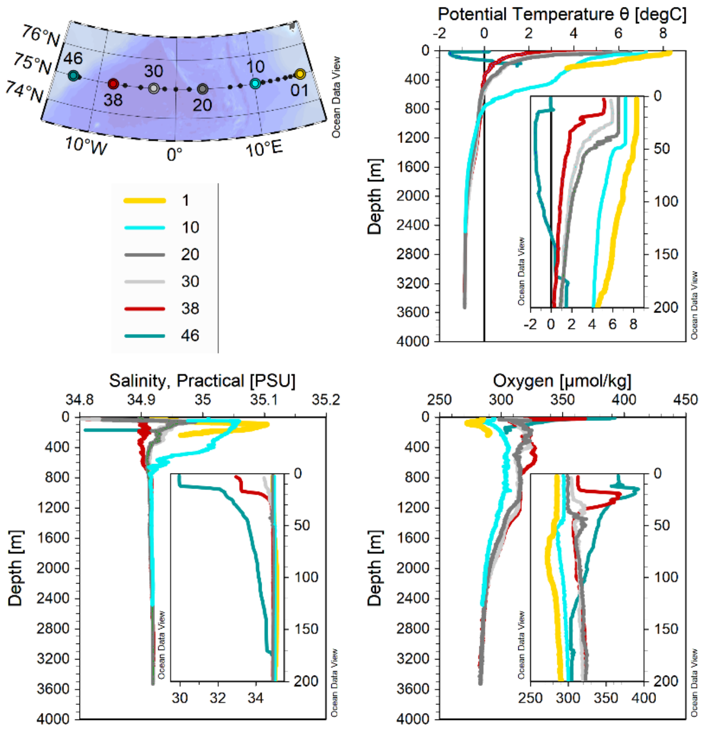

Figure S2. Full depth vertical profiles of potential temperature (°C), salinity (psu), and dissolved oxygen (µmol/kg) carried out at the biological stations 1, 10, 20, 30, 38, and 46 along the 75 °N transect. The inner panels show a zoom of the upper layer (0–200 m depth).

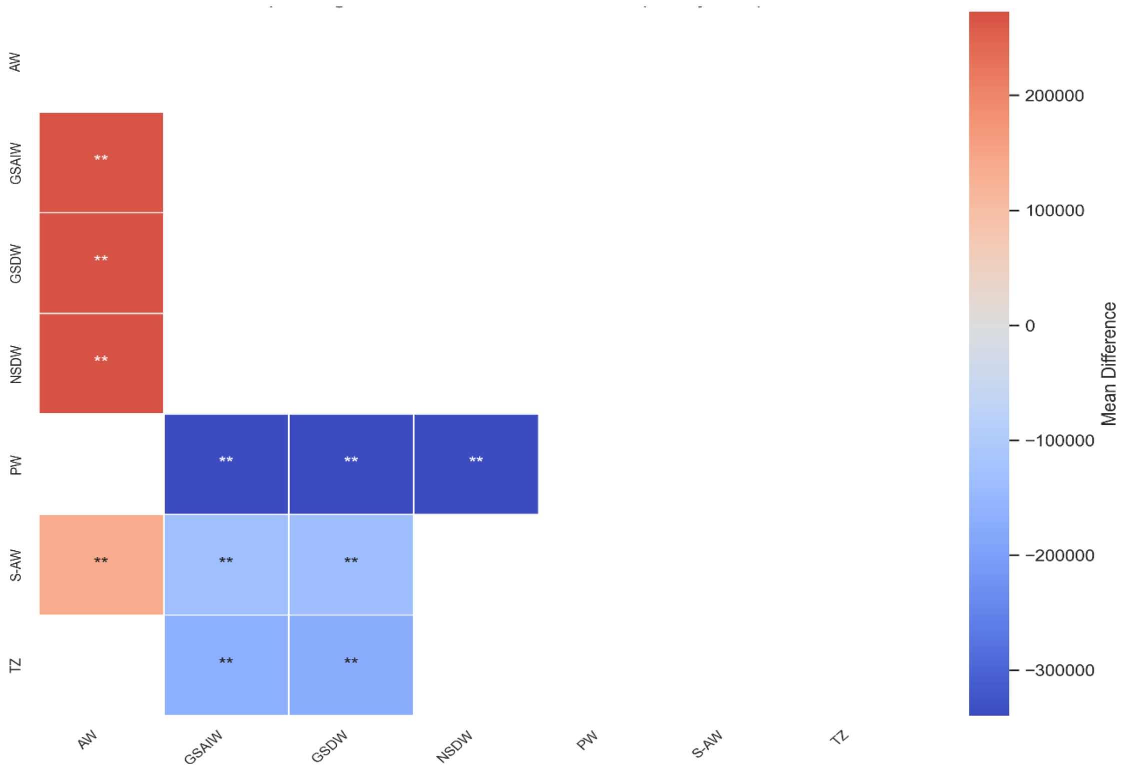

Figure S3. Heatmap showing pairwise mean differences among water mass groups based on microbial or environmental parameters, derived from Tukey's HSD post-hoc test following a significant PERMANOVA (Pseudo-F = 18.34, p < 0.001). Only significant comparisons are shown. Color intensity indicates the direction and magnitude of the difference (red = higher, blue = lower), and significance levels are annotated as follows: *p < 0.05, **p < 0.01, and ***p < 0.001. The results highlight substantial differences among water masses, particularly between Arctic and deep water layers.

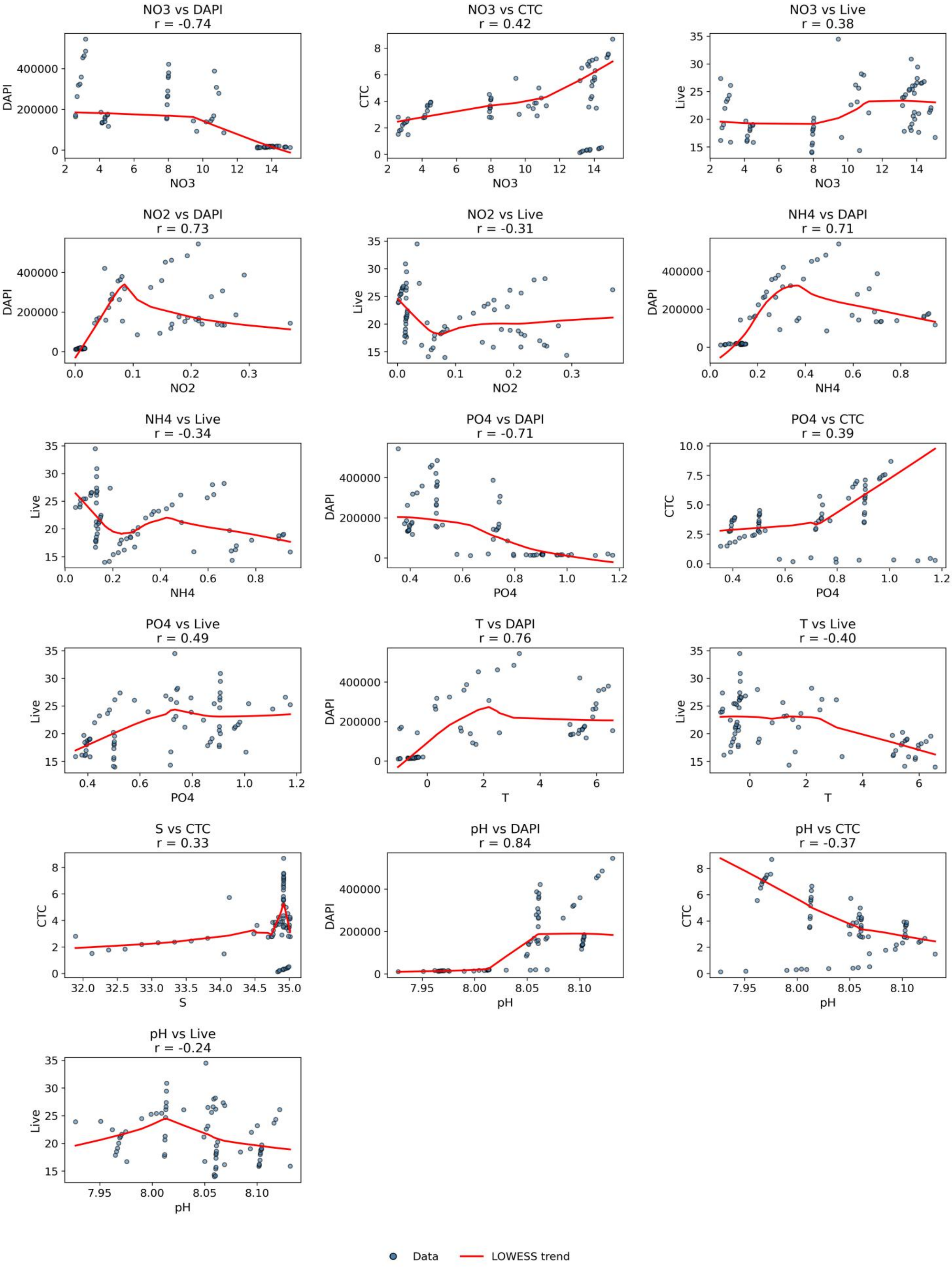

Figure S4. LOWESS (Locally Weighted Scatterplot Smoothing) curve illustrating the underlying trend in a noisy dataset. For each panel, a LOWESS (Locally Weighted Scatterplot Smoothing) curve was fitted (in red) to highlight the local trend in the data distribution. The Pearson correlation coefficient (r) is reported above each plot to indicate the direction and strength of the linear relationship.

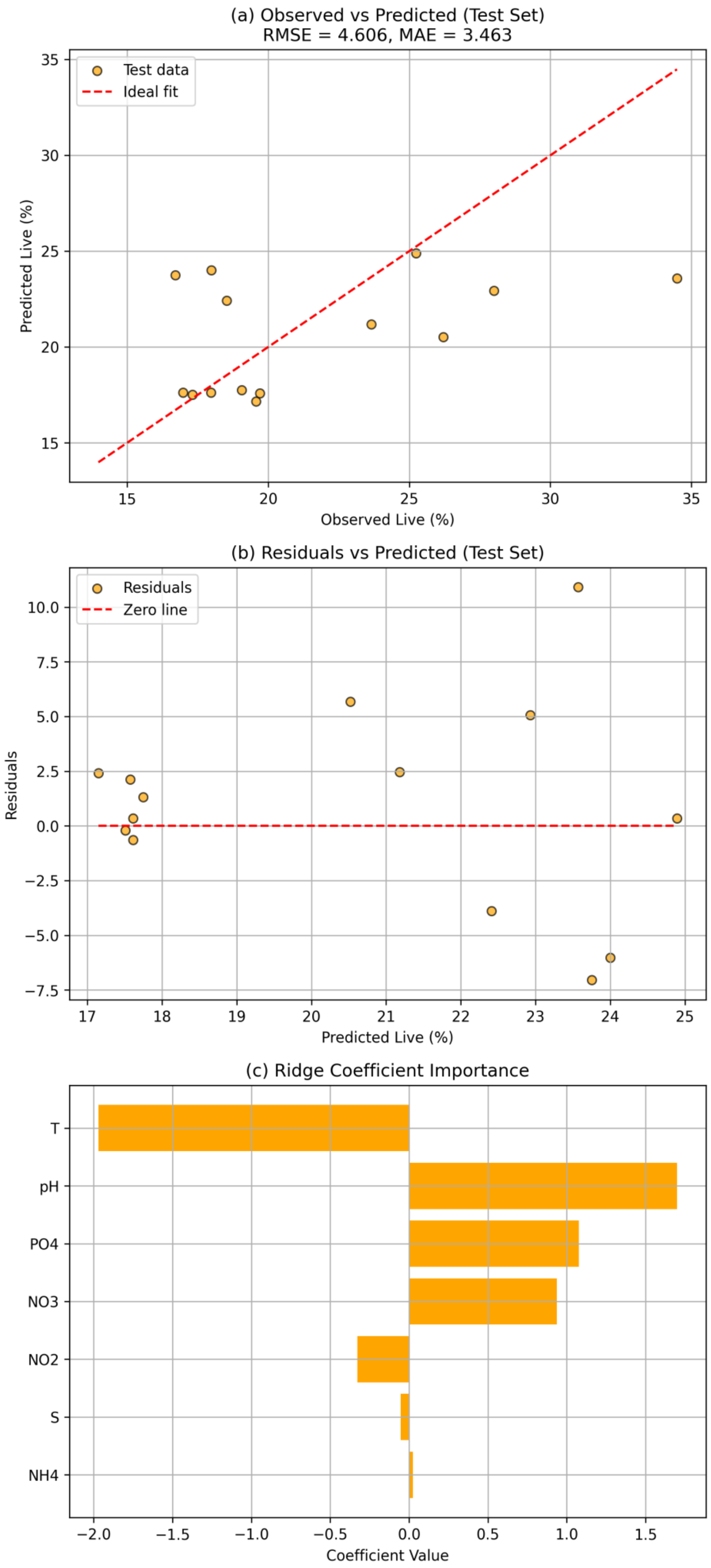

Figure S5. Evaluation of the Ridge regression model built to analyze the relationship between physico-chemical parameters (NO_2, NO_3, NH_4, PO_4, temperature, salinity, and pH)

and the percentage of live prokaryotic cells. (a) Scatterplot comparing observed and predicted values of live cells in the test dataset. The dashed red line represents the 1:1 reference line. Model performance on the test set yielded a root mean square error (RMSE) of 4.781 and a mean absolute error (MAE) of 3.688. (b) Residuals plotted against predicted values, with the red dashed line indicating zero residual. No evident patterns or heteroscedasticity were observed, suggesting acceptable model behavior. (c) Coefficient importance derived from the Ridge model, sorted by absolute value. The model identified PO_4 and pH as the most influential predictors, while other variables such as NO_3 and salinity had weaker effects. Coefficients were obtained using standardized variables and an optimized regularization parameter (α = 4.977) selected via cross-validation.

References

1. Schlitzer R (2025) Ocean Data View. Available from: http://www. odv.awi.de.

AIMS Press

© 2025 the Author(s), licensee AIMS Press. This is an open access article distributed under the terms of the Creative Commons Attribution License (https://creativecommons.org/licenses/by/4.0)
